# Towards a Molecular Systems Model of Coronary Artery Disease

**DOI:** 10.1007/s11886-014-0488-1

**Published:** 2014-04-18

**Authors:** Gad Abraham, Oneil G. Bhalala, Paul I. W. de Bakker, Samuli Ripatti, Michael Inouye

**Affiliations:** 1 Medical Systems Biology, Department of Pathology and Department of Microbiology & Immunology, The University of Melbourne, Parkville, Victoria 3010 Australia; 2 Department of Medical Genetics, University Medical Center Utrecht, Utrecht, The Netherlands; 3 Department of Epidemiology, University Medical Center Utrecht, Utrecht, The Netherlands; 4 Division of Genetics, Brigham and Women’s Hospital, Harvard Medical School, Boston, MA USA; 5 Institute of Molecular Medicine (FIMM), University of Helsinki, Helsinki, Finland; 6 Hjelt Institute, University of Helsinki, Helsinki, Finland; 7 Department of Human Genetics, Wellcome Trust Sanger Institute, Wellcome Trust Genome Campus, Hinxton, UK

**Keywords:** Coronary artery disease, Coronary heart disease, Genomics, Systems biology, Mendelian randomization, Metabolites, Network analysis, Molecular systems model

## Abstract

Coronary artery disease (CAD) is a complex disease driven by myriad interactions of genetics and environmental factors. Traditionally, studies have analyzed only 1 disease factor at a time, providing useful but limited understanding of the underlying etiology. Recent advances in cost-effective and high-throughput technologies, such as single nucleotide polymorphism (SNP) genotyping, exome/genome/RNA sequencing, gene expression microarrays, and metabolomics assays have enabled the collection of millions of data points in many thousands of individuals. In order to make sense of such 'omics' data, effective analytical methods are needed. We review and highlight some of the main results in this area, focusing on integrative approaches that consider multiple modalities simultaneously. Such analyses have the potential to uncover the genetic basis of CAD, produce genomic risk scores (GRS) for disease prediction, disentangle the complex interactions underlying disease, and predict response to treatment.

## Introduction

Coronary artery disease (CAD) is a significant impediment to good health and productivity worldwide. Globally, CAD is the leading cause of death with 7 million deaths in 2011 alone, accounting for 11.2 % of all deaths [1]. Unfortunately the impact of CAD is increasing, as the disease burden is projected to nearly double from 47 million disability-adjusted life years (DALYs) in 1990 to 82 million DALYs in 2020 [2]. However, this impact is asymmetrically distributed between developed and developing countries. CAD morbidity is projected to more than double in developing countries from 1990 to 2020, but only increase by 50 % in developed countries [3].

Development of CAD is a multidecade process of atherosclerotic formation and chronic inflammation that ultimately leads to angina, myocardial infarction (MI), and death [4]. It arises from complex interactions of a multitude of factors—both environmental and genetic. Risk factors, such as tobacco use, low physical activity, obesity, hypertension, hypercholesterolemia, and diabetes have long been known. The contribution of genetics has been suspected given the importance of family history for CAD in an individual’s current risk. This has been strengthened with data from Swedish twin studies demonstrating a substantially larger concordance rate for monozygotic twins compared with dizygotic twins and heritability of 0.57 for males and 0.38 for females [5, 6]. However, identifying the specific genetic changes that modify CAD risk has been, and still is, a challenge despite intense investigation in recent years.

## Uncovering the Genetic Basis of CAD

The publication of the Human Genome in 2001 offered an unprecedented launching pad for the understanding the genetic basis of diseases [7, 8]. Within 6 years, seminal papers were published that began to outline the genetic architecture of CAD. These were initially based on univariate genome wide association studies (GWAS), where frequencies of individual SNPs in those with CAD (cases) were compared with those without CAD (controls). Although these GWAS datasets were significantly larger than what researchers were used to at that time, the statistical approach of testing individual SNPs for association was rather straightforward. One of the first studies considered 1607 MI cases and 6728 controls with no history of CAD from an Icelandic population [9]. This genome-wide scan found and replicated a locus associated with MI whose top SNP yielded an odds ratio of 1.28.

In the same issue of Science, McPherson et al. also found a locus associated with CAD in the Ottawa Heart Study that validated in both the Copenhagen City Heart Study and Dallas Heart Study [10]. The top SNP in the locus increased the risk of CAD by 15 %–20 % in individuals who were heterozygous and 30 %–40 % in those who were homozygous. Analysis of CAD cases from the Wellcome Trust Case Control Consortium [11] and German MI Family Study revealed another SNP that was also strongly associated with CAD [12]. Each copy of the SNP allele increased CAD risk by 36 %.

Interestingly, these initial SNPs all mapped to chromosome band 9p21, identifying it as the first locus to harbor common variants for CAD [13]. This locus was also replicated in other populations including Italian, Japanese, Korean, and Indian, suggesting it may be a universal risk allele [14–16]. 9p21 does not code for any known proteins but the closest genes, within 150 kb, are *CDKN2A* and *CDKN2B*, which control cell proliferation and apoptosis. Moreover, targeted deletion of the noncoding interval of 9p21 in mice increased cardiac expression of *CDKN2A* and *CDKN2B* as well as vascular cell proliferation [17]. In addition, it has also been shown that 9p21 contains an abundance of enhancers and that the CAD risk SNPs disrupt STAT1 binding to 1 such enhancer thereby disrupting the interferon-γ response [18]. The mediator between 9p21 and downstream genes may be *ANRIL*, a noncoding RNA in the region, which can alter downstream gene expression [19, 20]. 9p21 has also been found to be associated with coronary calcification levels, abdominal aortic aneurysms, and intracranial aneurysms, suggesting a broader role for this variant in vessel function [21–24].

CAD has been associated with other loci in subsequent large-scale meta-analyses. A variant mapping to 6q25.1 increases CAD risk by 23 % per allele [12]. Notably, this SNP is located within the *MTHFD1L* gene, which can influence plasma homocysteine levels and affect risk of CAD [11]. New loci have also been identified using haplotype analysis, whereby proximal SNPs are grouped together and treated as a single unit, thus, enhancing the detection power. Re-analysis of the WTCCC dataset identified the 6q26-q27 locus, containing 4 SNPs within the *SLC22A3-LPAL2-LPA* gene cluster [25]. The *LPA* gene encodes for part of lipoprotein(a) (Lp(a)), whose plasma levels correlate with CAD pathogenesis [26, 27]. Analysis of the haplotype determined that it accounted for 15 % of Lp(a) plasma level variability. Soranzo et al. also used haplotype analysis to identify 12q24 as a CAD locus containing 2 SNPs yielding an odds ratio of 1.144 [28]. Interestingly, this locus also demonstrates considerably disease pleiotropy with type 1 diabetes, celiac disease, and hypertension.

## “Missing” Heritability

The initial univariate GWAS were able to identify common SNPs associated with CAD with low to moderate effect size [29]. However, these SNPs account for less than 10 % of CAD heritability, and it has been posited that the remaining unexplained heritability may be due to rarer variants with larger effects and/or undiscovered common variants with small effects [30–32]. These shortcomings suggested the need for larger data sets to allow for increased detection power. The CARDIoGRAM Consortium was formed to overcome these issues by amalgamating 14 GWAS with 22,223 CAD cases and 64,762 controls of European ancestry [33]. This meta-analysis identified 13 new loci, with each allele increasing CAD risk by between 6 % and 17 % [34]. The C4D Genetics Consortium performed another large-scale analysis, where 8424 cases of European ancestry and 6996 cases of South Asian ancestry, along with 15,062 controls were analyzed [35]. Five new loci were identified including 1 SNP of particular biological interest as the nearest gene, *PDGFD* (117 kb downstream), encodes the platelet-derived growth factor D protein. This protein is suspected to promote pathogenesis of atherosclerotic plaques and the SNP’s risk allele was associated with increased transcription of *PDGFD* in aortic media, aortic adventitia, and mammary artery. Together, both CARDIoGRAM and C4D recently identified 46 genome-wide significant loci, explaining a little over 10 % of CAD heritability [36]. Although many CAD SNPs are still to be discovered, more collaborative GWAS with larger and more diverse sample populations are needed to further explain the remaining “missing” heritability.

## Constructing Genomic Risk Scores

One of the potential benefits of genomic association studies is the use of an individual’s genomic profile to create a score capturing the risk of developing CAD. Currently, the Framingham Risk Score (FRS) is a powerful tool that incorporates a patient’s age, sex, cholesterol, smoking status, blood pressure, and diabetes status to generate a 10-year CAD risk prediction [37]. However, it can be the case that a majority of new CAD cases appear in individuals who are not in the highest FRS risk group [38]. Given that many of the FRS factors are dependent on age and/or have a genetic component, the use of genetic variants may allow for earlier and more efficient identification of those at risk for CAD before changes in other risk factors like blood pressure or plasma lipid levels occur. Therefore, a genomic risk score (GRS), comprised of the increased risk associated with genetic variants, can be calculated and used to stratify patients into different risk categories, as is done with the FRS; early stratification being crucial for maximum benefit from lifestyle modification or therapeutic intervention to stymie pathogenesis.

As an initial investigation of the usefulness of a GRS with regard to individual risk prediction, Ripatti et al. built a 13-SNP GRS for CAD and tested its utility in a prospective study of 30,725 individuals of European ancestry free of cardiovascular disease [39•]. They found that patients with a GRS in the top 20 % had a 66 % increased risk (95 % CI: 35 %–104 %, *P* = 10^-10^). In another study, a very similar 13-SNP GRS was tested in 3014 individuals from the Framingham Heart Study with an 11-year median follow-up [40]. Analysis in this population found that each risk allele had a hazard ratio of 1.07 (95 % CI: 1.00–1.15, *P* = 0.04). Although this GRS did not improve prediction of CAD over the FRS, it did associate strongly with high coronary artery calcium levels, a marker for subclinical atherosclerosis, suggesting that these SNPs may be predictive of atherosclerotic plaque progression.

In another prospective study of 4818 Caucasian males [41], a 15-SNP GRS improved net reclassification (6.5 %, *P* = 0.044) and discrimination (1.11 %, *P* = 0.048) over FRS, indicating that there was a marginal improvement of identifying those at risk for CAD [42]. The largest benefit of the 15-SNP GRS was realized when analysis was restricted to men between 50 and 59 years, where a 2.8 % (*P* = 0.0038) discrimination improvement over FRS occurred. Analysis of a 28-SNP GRS in a Finnish-based prospective cohort of 24,124 individuals also showed an improvement over FRS [43]. In this study, FRS provided a discrimination index of 0.851, while adding the GRS improved the index to 0.856 (*P* = 0.0002). Moreover, addition of the GRS helped reclassify 12 % of the individuals as high-risk, potentially identifying a group of patients that would benefit from early targeted healthcare. The value of a GRS also extends beyond individual risk prediction as it also captures a "best guess" of the genetic architecture of disease/trait, thus, genetic overlaps between CAD traits can also be assessed [44].

Although it is still early days, these GRS studies point to the potential use of SNPs in identifying and classifying individuals as high- and low-risk for CAD. It is also clear that GRS's based on lead SNPs from each associated locus use only a small fraction of the potential for prediction and more elaborate strategies and statistical methods, such as supervised learning, are needed to optimize the genomic predictions [45]. Further analyses using large numbers of genetic variants are needed to determine the effectiveness of a GRS as a screening tool and indicator for intervention, especially in or before the early stages of pathogenesis where FRS variables like age, cholesterol, and blood pressure are less predictive and lifestyle modifications can have a greater impact.

## Network Approaches to Understand CAD Etiology

Despite the success of GWAS, these results only provide 1 piece of the puzzle. It has been challenging to interpret the individual SNP associations in terms of the underlying biological pathways, and therefore, current knowledge of disease etiology is still limited. To have a phenotypic effect, true causal genetic variants must exert their influence through other mediators, such as changes in the structure of protein encoded by the gene (eg, nonsynonymous variants) or changes in regulatory elements affecting the binding affinity of transcription factors, thus, affecting the gene’s RNA levels and presumably the levels of the encoded protein. Data about these biological phenomena were not available in many early studies, limiting the ability to decipher these complex effects. To address this shortcoming, studies now aim to combine various sources of systems level information, such as genomic, transcriptomic, and metabolomic data. Because both gene expression and metabolites are known to be influenced by genetic variation, joint investigation of these genetic effects on gene expression and metabolite levels and on clinically-relevant phenotypes such as atherosclerosis affords insight into how the genetic effects on disease are mediated [46•].

Beyond the ability to examine multiple sources of biological data at once, another important conceptual advance has been the shift toward examining genes as part of networks, such as transcriptional regulation networks [47], rather than considering them in isolation. A network-based analysis has the power to better model these biomolecular relationships and their potential role in disease. The network is the context: in some cases, the individual gene associations may be weak but subtle global trends may reveal themselves when examined at the level of multiple related genes.

**Fig. 1:**
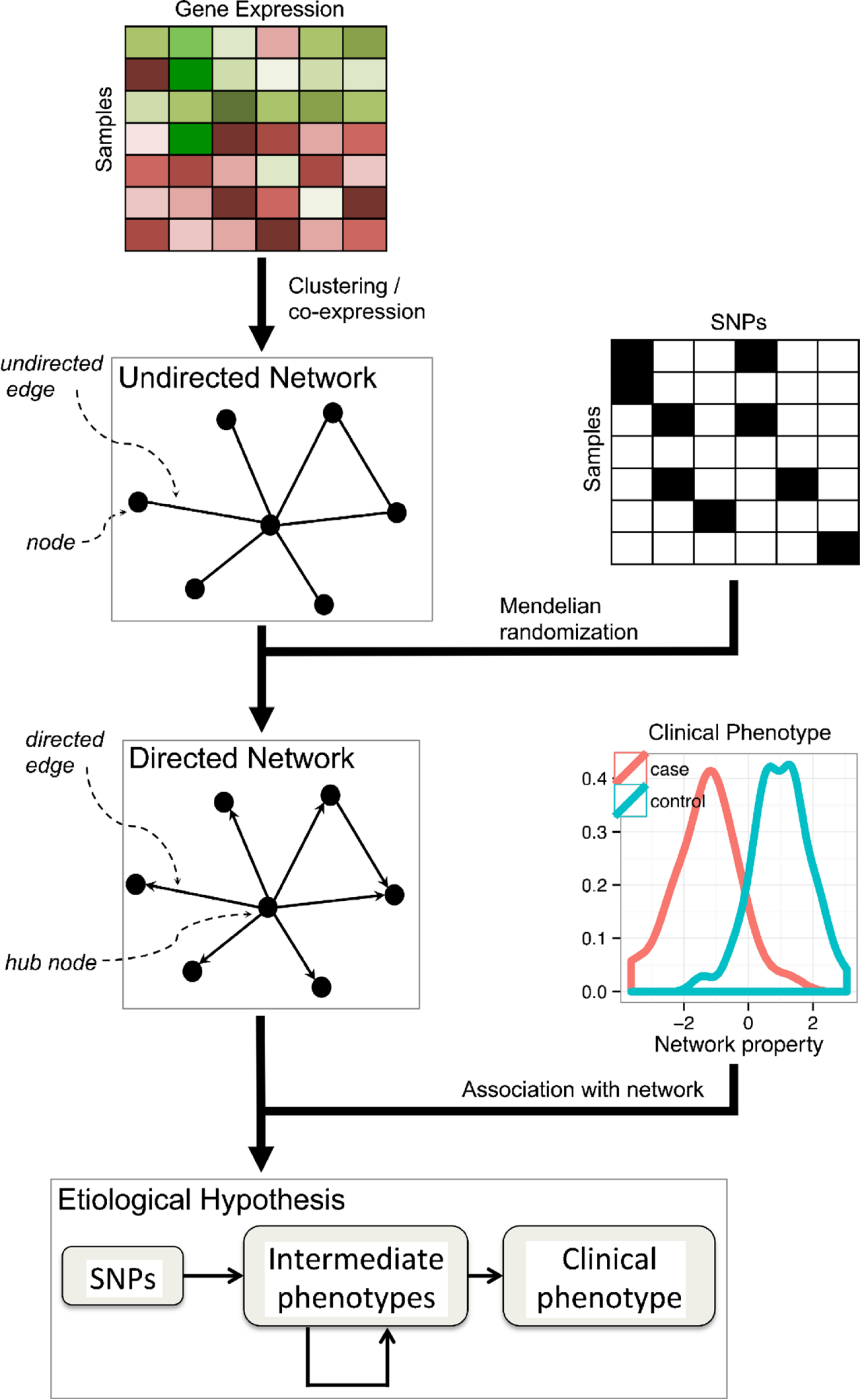
Network-based analysis of omic data to model the processes connecting genetic variation to disease

In a formal mathematical sense, a network consists of nodes (also called vertices) connected by edges (Fig. 1). Typically, nodes represent some discrete entity, such as a gene, protein or metabolite, whereas edges represent relationships between entities. The edges may be *undirected*, representing a symmetric relationship such as “gene A is correlated with gene B” or *directed*, representing an asymmetric relationship such as “gene A upregulates gene B”. The edges need not represent purely physical relationships such as interactions; they may simply represent statistical effects such as correlation. One of the earliest and simplest applications in network analysis has been visualization of complex relationships between biological entities such as genes and genetic variants, allowing the researcher to construct a mental image of the underlying etiology. However, as the data becomes larger, more complex, and multi-faceted so the networks become less interpretable, requiring more sophisticated approaches for visualization and analysis in order to extract biological meaning from them.

Researchers have long known that information sources such as transcriptomics can offer valuable insights into CAD etiology [48, 49], as circulating leukocytes link organ systems in mediating the atherosclerotic process [50], however, integration with other data sources including genetic and metabolite variation has only been possible relatively recently with the wide-spread use of high-throughput assays. A summary of recent examples of gene networks that have been linked to CAD etiology is given in Table 1. One of the first examples of network-driven models of complex disease is that by Chen et al. [51], who examined genotypes, gene expression, and metabolite data from BxH-cross mice. They constructed networks of co-expressed genes and identified highly-connected components (modules) of these networks that were associated both with genetic variants and with several intermediate phenotypes related to obesity, diabetes, and atherosclerosis, such as abdominal fat mass, weight, plasma insulin, free fatty acids, total plasma cholesterol, and aortic lesion size. Using a statistical approach based on Mendelian Randomization [52] (see below), some of these sub-networks were then postulated as causal mediators between genetic variants and the disease-related traits. One of the modules with the strongest associations that was also supported to have a causal effect on metabolic traits was the macrophage-enriched metabolic network (MEMN) consisting of 1406 genes, out of which 375 genes were estimated to be causal of obesity-related traits. From these genes, it was hypothesized that *Lpl* and *Lactb* caused obesity and that *Ppm1l* was a causal driver of phenotypes related to metabolic syndrome, predictions which were subsequently validated in mice [53]. Significantly, the MEMN network together with its link to obesity were also replicated in human adipose tissue [54]. Beyond the specific findings of these studies, these results highlight the power of integrating several sources of information and of considering networks rather than genes in isolation to uncover some of the biological processes connecting genetic variation with observable phenotypes.

**Table 1:** Molecular networks relevant to coronary artery disease

Name	Source	Main associations	Biological interpretation	Ref
Macrophage-enriched metabolic network (MEMN)	B × H mouse cross, multiple tissues	Obesity, diabetes, atherosclerosis, total cholesterol levels, and HDL levels	Network represents an inflammatory response driven by genetic variation and affecting disease phenotypes, in liver and/or adipose tissue.	[51]
Lipid-leukocyte (LL) module	Human population-based cohort, whole blood	Various metabolites (HDL, VLDL, glycoproteins, isoleucine, and others) and immune response markers (IL-1ra, CRP, and heavy molecular weight adiponectin)	Module implicates acute inflammatory cells (mast cells and basophils) as reactive to various metabolites. Inverse relationship between module expression and its density.	[69,70]
Combined inflammatory pathway	Human cohort enriched for low familial HDL-C, adipose tissue	VCAM1 levels and SNPs predictive of low HDL-C	Pathway represents an inflammatory link between genetic variation and HDL-C levels and indicates that HDL levels may be regulated by inflammatory processes.	[62]
Two differential modules (case and control)	Human CAD case/control study, whole blood	Control module enriched for SNPs predictive of CAD.	Differential pathways represent 2 modes of regulation acting in CAD vs non-CAD individuals, and indicate that B-cell immune pathways may be causally driving lipids levels and risk of CAD.	[63•]

*CRP* C-reactive protein, *HDL* high density lipoprotein, *HMWA* high molecular weight adiponectin, *IgE* immunoglobulin E, *IL-1ra* interleukin 1 receptor antagonist, *VCAM1* vascular cell adhesion molecule 1, *VLDL* very low density lipoprotein

## Mendelian Randomization for Predicting Causality

Network analyses tend to rely on statistical association, such as correlation, to infer the network structure from observational data. In order to identify new drug targets and interventions that can reduce disease risk, we must identify which factors cause disease and, which are merely associated with it, as association does not necessarily imply causation [55]. For example, if a metabolite is observed to be associated with disease, one cannot say that the metabolite is causal of the disease based on this observation alone. Such a claim can only be made using methods such as interventional experiments where the metabolite level is manipulated and the resulting effect on disease is observed. Yet, in some cases, statistical analyses of genomic variation may be able to determine which relationships are causal. Genetic variation is unique in that an individual’s alleles are assigned randomly during meiosis (assuming nonassortative mating with respect to the phenotype of interest) and are largely fixed for the life of the individual. Consequently, genetic variants can be viewed as natural perturbations of the system (Mendelian Randomization [56, 57]), allowing us to interpret a genetic-phenotypic association as causal in 1 direction, namely genetic variant causes phenotype. Leveraging this causal information through statistical frameworks such as instrumental variables (IVs) allows one to infer whether other associations with disease are indeed causal or simply correlations. Such an approach has provided evidence that increases in plasma HDL cholesterol do not lower the risk of myocardial infarction despite the well-documented strong negative correlation between the 2 in observational studies, thus, drug targeting of HDL-C for CAD treatment may not be a successful strategy [58•]. Applied on a larger scale, statistical methods based on the principles of Mendelian Randomization approaches can help orient the edges in an undirected gene or metabolite network (inferred from associations), generating directed networks representing putative causal structures [52, 59, 60] that can later be tested experimentally either in laboratory models or randomized controlled trials in humans.

## Integrative Omics to Decipher the Role of Inflammation in CAD

In humans, large-scale multi-omic datasets are being increasingly utilized to better elucidate the biological pathways responsible for the known links between inflammation and CAD [61]. Laurila et al. [62] analyzed Finnish individuals assayed for transcriptomics in fat tissue, HDL lipidomic profiles, and genotypes, in order to dissect the genetic contribution to levels of plasma high-density lipoprotein cholesterol (HDL-C), as HDL-C levels are known to be negatively associated with risk of CAD. The analysis compared multi-omic profiles of individuals at the extremes of HDL distribution and highlighted the role of the *HLA* region and of inflammation pathways in controlling HDL-C levels and a wide range of differences in both adipose transcriptome and lipidomic profiles. Another analysis of CAD case/control individuals from the Framingham Heart Study [63•] revealed differences in co-expression patterns of genes between cases and controls; these *differential modules* were found to be enriched for quantitative trait loci (QTLs) that affect CAD risk, including a module enriched for B-cell immune genes. By integrating various data sources, including protein-protein interaction (PPI) networks and gene networks derived from other studies, they found several regulatory genes, dubbed *key drivers*, exhibiting strong effects on these modules. One such gene was *TNFRSF13C*, which is known to affect aortic root atherosclerosis, thereby providing support for the hypothesis that changes in coregulation of B-cell-related gene networks, caused by such drivers, was partly responsible for increases in CAD risk.

Expanding beyond the small set of metabolites traditionally associated with CAD (eg, total LDL/HDL cholesterol or triglycerides), characterization of a wide-range of metabolite levels has become increasingly important in understanding of etiology CAD and related metabolic diseases [64, 65]. Atherosclerotic plaques themselves are heterogeneous and composed of multiple immune cell types and a wide variety of fats, lipoproteins, and other metabolites [66, 67]. Thus, there is a need to include metabolomic variation together with genetic and transcriptomic effects. Such analyses have now become possible with technological advances in ^1^H NMR and high-resolution mass spectrometry, which routinely measure levels of hundreds of metabolite species in large human cohorts. For example, a recent metabolomic GWAS of 216 serum metabolite measures in over 8300 Finnish individuals [68] identified 31 loci with genome-wide significant associations to metabolite levels. Integration of genomics, transcriptomic, and metabolite data in a large Finnish cohort led to specific evidence for the role of inflammation in CAD through the identification of the Lipid-Leukocyte (LL) module [69], a network of highly co-expressed genes related to the acute inflammatory response, which was inferred to be reactive to lipid levels through causal inference methods [59]. This analysis was later extended to assess a wide-range of metabolite species, revealing the effect of specific sub-species on the module’s coherence, indicating that the degree of co-expression in the module was significantly associated with metabolite levels such as linoleic acid and various LDL and HDL particles [70]. Later, by integrating serum metabolomics data with genetic variation and transcriptomic data in humans and in mice, Inouye et al. [46•] used a powerful data-driven multivariate approach to identify 11 metabolic networks and detect 7 previously-unknown loci associated with serum metabolite levels, notably *SERPINA1* and *AQP9*. Transcriptional data was then used to show that *AQP9* expression in murine liver was associated with the size of atherosclerotic lesion and, in humans, both *AQP9* and *SERPINA1* expression in arterial tissue was substantially upregulated in plaques, providing a potential explanatory link between genetic variation and a phenotype relevant to disease progression.

## New Data Sources to Model Disease

Apart from genetic, transcriptomic, and metabolomic data, another rich source of information is the microbiome, the characterization of the diverse set of microbial communities inhabiting the human body, which are increasingly being recognized as important factors in obesity and atherosclerosis [71–73]. Recent evidence points to the presence of oral pathogens such as Chryseomonas, Veillonella, and Streptococcus in atherosclerotic plaques and, thus, potentially contributing to the inflammatory process [74, 75]. A recent example of one such integrative study involving the gut microbiome, genetic variation, and gene expression in inbred mice strains (Hybrid Mouse Diversity Panel) [76] across several time points revealed the complex interactions between these components in contributing to obesity. Further, the effects of high-fat/high-sucrose diets on the composition of gut bacterial communities was modulated by the specific genetic makeup of each mouse strain, leading to down-stream effects on metabolism and eventual risk of obesity.

Another potential source of useful information is epigenetics, which describes both epigenetic marks and noncoding RNAs (ncRNAs) [77, 78]. Many epigenetic marks are thought to be reset during early embryogenesis; however, some epigenetic marks may still be passed between generations [79], and marks may be modified in response to environmental exposures such as smoking [80]. Some epigenetic effects relevant to CAD include homocysteine-induced methylation in vascular smooth muscle cells, contributing to atherosclerosis [81], and the ncRNA microRNA-33 found to regulate cholesterol homeostasis [82]. Further large-scale studies will be required in order to assay genome-wide methylation status and ncRNA expression levels in concert with other data sources.

## Future Directions

Apart from incorporating novel sources of biological variation, an important factor not considered in many studies is time itself: the biological processes underlying cardiac and metabolic disease are dynamic, all while interacting with a complex array of environmental and genetic effects. CAD may take several decades to manifest clinically, and is typically preceded by subclinical phenomena. Clearly, this disease progression can be influenced at various stages by external interventions such as life-style changes (diet and exercise) and medication such as statins, and disease trajectories vary widely between individuals. Hence, to better understand these processes larger and more detailed repeated measures data will be required. Such experiments present their own unique challenges in analysis and interpretation [83, 84]. A recent multi-omic study examined whole-genome sequencing, RNA sequencing, proteomics, metabolomics, ncRNA, and auto-antibody data measured in blood components from 1 individual over a 14 month period [85], revealing in detail the correlation of multiple body systems like inflammatory and insulin response pathways to viral infection and early onset of type 2 diabetes. This study provides a glimpse into what will become more common, with costs of technology reducing to a level enabling such detailed measurements over much larger cohorts and longer time-scales. Similar studies with much larger sample sizes based on individuals with various disease states will be necessary to be able to draw robust conclusions about the pathogenesis of CAD and improve preclinical models.

To complement temporal dynamics, spatial effects will need to be considered as well, that is, heterogeneity in expression across tissues [86, 87]. Studies that consider only 1 tissue type, typically blood and its components, will not be able to detect such variation. This will require assays of multiple tissue types, both to capture coordinated processes happening in all tissues and to localize effects that occur only in specific tissues. One such effort is the Genotype-Tissue Expression (GTEx) project [88], which aims to conduct a wide-ranging survey of gene expression and its associated genetic variation across multiple human tissues.

In addition to the important goals of predicting those individuals that are at high risk or disease and developing a deeper understanding of disease etiology, another major avenue of research where systems and network approaches may have an impact is pharmacogenomics, that is, the study of how genetic variation influences each individual’s response to medication. Both drug response and drug metabolism can be considered as phenotypes, and many of the existing analysis methods are applicable. This will be useful for tailoring more specific sets of medication to each individual, matched to their genomic profile [89, 90]. The clinical utility of pharmacogenomics approaches has so far been rather limited [91]. However, testing for loss-of-function variation in the gene *CYP2C19* (part of the P450 family of enzymes) is now recommended for informing the use of the antiplatelet drug clopidogrel in individuals with acute coronary syndrome (ACS) undergoing percutaneous coronary intervention (PCI) [92] because such variants may adversely affect platelet activity and increase the risk of cardiovascular events.

## Conclusions

CAD and related diseases are complex phenotypes and are the result of interplay amongst a multitude of genetic and environmental effects together with an important inflammatory aspect. Initially, analyses tended to be restricted to only 1 type of high throughput data, such as GWAS considering association of disease with genetic markers, or only considering blood biomarkers for disease progression. Although such focused analyses have proven insightful, they provide limited information about a narrow aspect of the disease, while ignoring the complex interactions between these different processes. The advent of multi-omic studies, which concurrently analyze genetic variation, transcriptomics, metabolomics, and others sources of information over hundreds or thousands of individuals, has begun providing deeper insight into the underlying mechanisms responsible for observed phenomena, such as the gene networks responsible for the known link between inflammation and disease and the various feedback loops amongst genetic variation, microbial communities, metabolism, and ultimately disease.

Hence, the coming major challenges will firstly be collection of large-scale multi-omic samples across thousands of individuals in order to achieve the statistical power to detect the multitude of interacting effects in CAD etiology. Second, novel statistical and computational methods will be required to effectively combine these different information sources into a coherent model of disease, with the aim of generating plausible biological hypotheses that can be tested in animal models and clinical trials. Third, the testing of these models will need to rigorously evaluate the specific reliability of the models as well as their modes of failure so that refinements to the model can be made. Meeting these challenges will result in greater understanding of CAD etiology and, if successfully implemented, translate into better clinical outcomes.
